# Cooperative carotid artery centerline extraction in MRI

**DOI:** 10.1371/journal.pone.0197180

**Published:** 2018-05-30

**Authors:** Andrés M. Arias-Lorza, Daniel Bos, Aad van der Lugt, Marleen de Bruijne

**Affiliations:** 1 Biomedical Imaging Group Rotterdam, Departments of Radiology and Medical Informatics, Erasmus MC, Rotterdam, The Netherlands; 2 Department of Epidemiology, Erasmus MC, Rotterdam, the Netherlands; 3 Department of Radiology and Nuclear Medicine, Erasmus MC, Rotterdam, the Netherlands; 4 Image Section, Department of Computer Science, University of Copenhagen, Denmark; Worcester Polytechnic Institute, UNITED STATES

## Abstract

Centerline extraction of the carotid artery in MRI is important to analyze the artery geometry and to provide input for further processing such as registration and segmentation. The centerline of the artery bifurcation is often extracted by means of two independent minimum cost paths ranging from the common to the internal and the external carotid artery. Often the cost is not well defined at the artery bifurcation, leading to centerline errors. To solve this problem, we developed a method to cooperatively extract both centerlines, where in the cost to extract each centerline, we integrate a constraint region derived from the estimated position of the neighbor centerline. This method avoids that both centerlines follow the same cheapest path after the bifurcation, which is a common error when the paths are extracted independently. We show that this method results in less error compared to extracting them independently: 10 failed centerlines Vs. 3 failures in a data set of 161 arteries with manual annotations. Additionally, we show that the new method improves the non-cooperative approach in 28 cases (*p* < 0.0001) in a data set of 3,904 arteries.

## Introduction

Centerline detection of the carotid artery in MRI is important to analyze its geometry [1], and to provide input for segmentation [1–3] and registration methods of the carotid artery [4, 5].

There are two main type of approaches to extract vessel centerlines using different image modalities: global optimization methods based on minimum cost paths, and local approaches [6]. Local methods include tracing medial axis from inscribed disks or spheres [7, 8], finding the centers of intensity ridge traversals [9, 10], and localizing local maxima from filter outputs [11, 12].

As minimum cost paths methods are based on global optimizations, they could result in robust centerlines [1, 13–16]. Often the carotid artery centerline is detected as two independent paths [1, 7, 17], one for each of the two arteries that originate from the Common Carotid Artery (CCA) at the bifurcation. These two arteries are the Internal Carotid Artery (ICA) and the External Carotid Artery (ECA). Using minimum cost path approaches, each path is defined as the minimum cost path between two points, where the cost is the output of a function that should be low at the center of the artery and high elsewhere. In this work, the centerlines are represented as minimum cost paths. There are two main techniques to find the minimum cost paths: graph approaches (Dijkstra algorithm [18], A* and F* algorithms [19]) and continuum approaches (fast marching [20]). In this work we use fast marching as it suffers less from metrication errors [21], and it provides the possibility to consider the anisotropic characteristic of the MRI images [20, 22].

**Fig 1:**
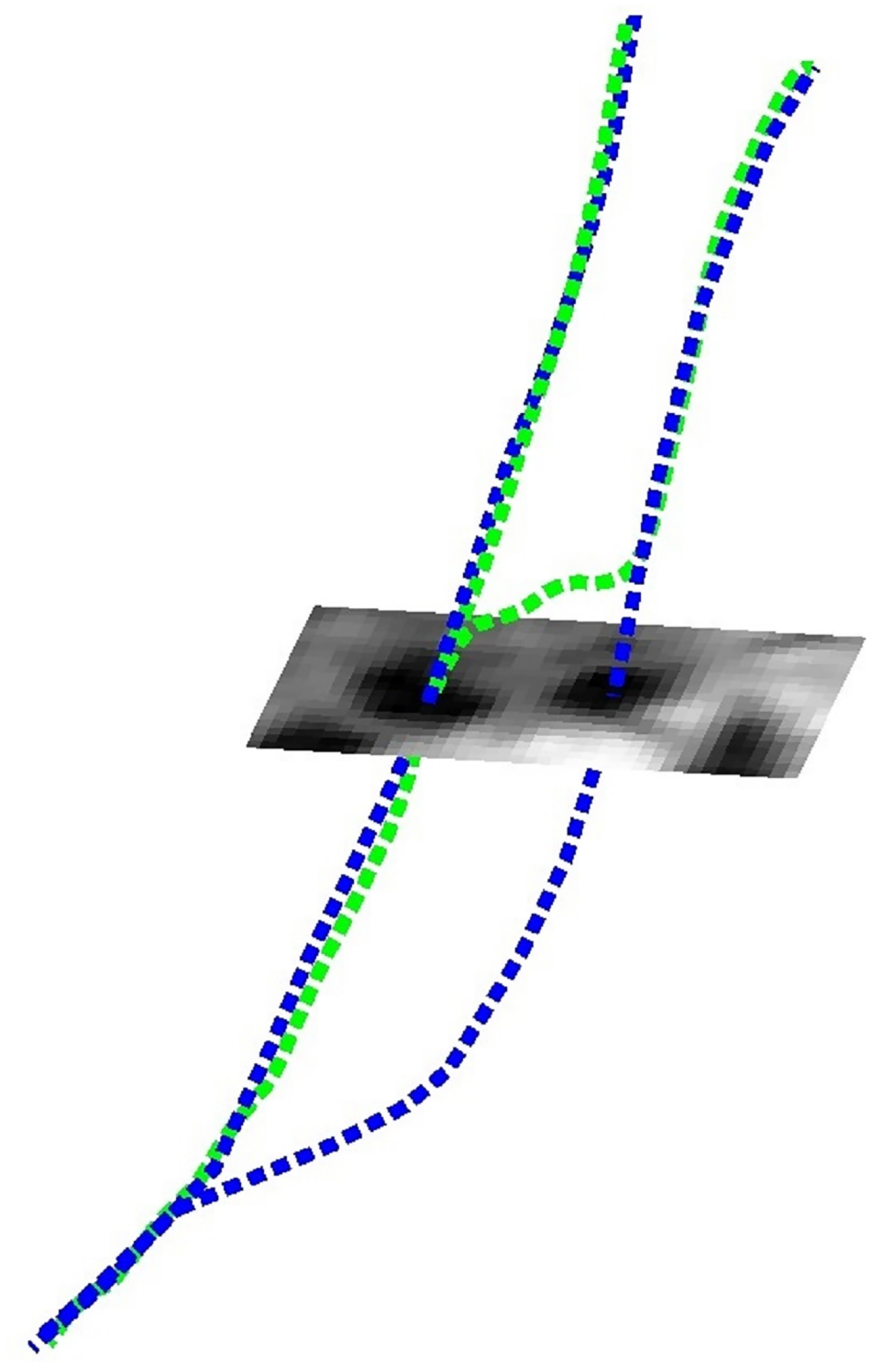
Centerline detection at the carotid artery by finding two independent minimum cost paths. The blue curves represent the manually annotated centerlines, and the green curves are the automatically detected centerlines. The centerlines are overlaying an MRI cross-section.

Defining a good cost function is a difficult task especially at the bifurcation. Generally, this cost at each image position is a function of the intensity and/or the surrounding shape [1, 6, 13–16], where the surrounding shape is estimated using Hessian eigen-analysis or medialness filters [12, 23]. However the intensity inside the artery presents a complex distribution along the vessel which is affected by artifacts, also surrounding structures may have similar intensities. Additionally vessel detector filters based on Hessian or medialness filters mainly work well on cylindrical shape structures. This can cause the centerline to present errors especially at the bifurcation, where the shape is not cylindrical. A common problem in carotid centerline extraction from MRI is that the centerline traced from either ICA or ECA jumps to the more clearly visible artery. An example is given in Fig 1.

To solve this problem, better cost functions that make use of features that describe well the artery shape and the intensities distribution inside the artery could be defined. Another solution can be to define a cost function that takes the anatomy and geometry of both arteries (ICA and ECA) into account. This could potentially prevent errors as shown in Fig 1. In this paper, we propose a method to cooperatively extract both ICA and ECA centerlines where the cost at each path considers the geometry of the neighbor artery.

The problem of jointly extracting multiple centerlines has been addressed before in medical imaging. In [24] a method to track the centerlines of abdominal vessels in 3D ultrasound is presented. They use several manually annotated points at the common, bifurcation, and at the bifurcated vessels; then the points are connected by straight lines which start an active contour evolution (snake) leading to the centerlines. This is an interesting approach and may work well if the initialization represented by the connected straight lines is inside the artery. However in many curved vessels the straight lines could be outside the artery. Other methods find rough segmentations of all the vessels of interest, and subsequently a skeletonization results in the centerlines [25]. This method may work if the segmentation is relatively accurate. However, the segmentation of the carotid artery in MRI is difficult and generally requires elaborated methods that use initializations based on centerlines [1–3]. Another method is [26], which obtains a complete tree of vessel centerlines by matching appearance models based on fitting cylindrical patterns. This is an interesting method as it is fully automatic; however they reported errors at the bifurcation for complex shapes.

Joint extraction of several minimum cost paths has been addressed before for multiple path planning where interaction and constraints in the paths are considered [27–29]. We find especially interesting the method presented in [27] where the cost of each path is defined by the path distance to the target and a constraint around the neighbor paths. This principle could be used to find multiple centerlines where the cost of the paths is a combination of the traditional cost based on intensity and shape features, and a constraint around the neighbor centerline. This should prevent the centerlines following the same paths either at the ICA or ECA as described in Fig 1 leading to more accurate centerlines at the bifurcation.

In this paper, we use a similar approach to find carotid artery centerlines. We define the centerlines as minimum cost paths where a constraint around the neighbor path is included. The cost is defined similarly to [1]. Further we solve the minimum cost paths using anisotropic fast marching as in [22] to consider the inherent anisotropic characteristics of MRI. Smoothness is also enforced by constraining the length of the path as in [21]. To evaluate the method, we compare the automatically extracted centerlines to manually annotated centerlines. In a large data set of 3904 arteries where manually annotated centerlines are not available, visual inspection of the results were performed in all cases where the conventional and proposed approach yield different results.

## 1 Method

First we describe how to obtain a representative cost image using only image information. Subsequently, we describe the anisotropic fast marching to extract minimum cost paths. Finally, we present the cooperative centerline extraction method which includes in the cost a constraint region derived from the likely position of the neighbor centerline.

### 1.1 Method overview

1. Obtain cost image which is low at the center of the artery and high elsewhere.
2. From seed points at CCA, ICA, ECA, and the bifurcation, apply anisotropic fast marching to get minimum cost paths between points.
3. As the paths may coincide after the artery bifurcation, we apply the new cooperative centerline extraction method to force the centerlines to follow different paths beyond the bifurcation.

### 1.2 Cost image

In [1] the cost is defined by a combination of the inverse of a multi-scale medialness filtering $m:R^{3}\to \left[0,1\right]$ [12] and inverse of artery lumen intensity similarity $s:R^{3}\to \left[0,1\right]$ [1]. The medialness filter gives a high output at the center of circular shapes, while the lumen intensity similarity metric is high when the intensity is similar to the mean intensity inside a Region Of Interest (ROI) around the set of seed points. These ROIs are spheres centered at the seed points with radius of 3.5*mm* for the CCA, and 2.5*mm* for ICA and ECA. As in [1] the cost function at voxel position **x** is given by:

$$\begin{array}{c} p\left(x\right)=\frac{1}{\epsilon +m{\left(x\right)}^{\alpha }s{\left(x\right)}^{\beta }}, \end{array}$$ (1)

where *ϵ* is a small positive value to prevent singularities, and the parameters *α* and *β* control the contribution of the medialness filter and the lumen similarity metric. In the case of multispectral MRI, the maximum output of the medialness and the intensity similarity terms over the different MR sequences is taken to compute *p*.

### 1.3 Minimum cost path using anisotropic fast marching

Given the cost image *p*, the minimum cost path $C^{*}:R\to R^{3}$ between two points is defined as the path *C* minimizing the total accumulated cost. In the continuous space the total accumulated cost by the path is defined by:

$$\begin{array}{c} \int_{C}(p(C(s))+\omega )ds=\int_{C}\tilde{p}\left(C\left(s\right)\right)ds, \end{array}$$ (2)

where *s* is the arc-length parameter. As in [21] to enforce smoothness in the path, a constant *ω* is added to *p*. By this, the maximum path curvature is inversely proportional to *ω* [21].

To minimize Eq 2, first the minimal action map $U:R^{3}\to R$ associated to the starting point $p_{0}\in R^{3}$ has to be defined. This minimal action map is defined as the minimum total cost to reach each point in the map, which satisfies the Eikonal equation represented by:

$$\begin{array}{c} \left\|\nabla U(x)\right\|=\tilde{p}\left(x\right), \end{array}$$ (3)

where *U*(**p**_0_) = 0. Subsequently, after having the minimal action map *U*, the minimum cost path C* between **p**_0_ and the end point $p_{1}\in R^{3}$ is obtained by backtracking the vector field ∇*U*(**x**) from **p**_1_ to **p**_0_.

Fast marching [20] is a numerical method to efficiently solve Eq 3 in the image space. As the images we use are 3D anisotropic MRI images, we use the 3D anisotropic fast marching algorithm based on [22]. Here *U*(**x**) is the solution to the quadratic numerical approximation of Eq 3 given by:

$$\begin{array}{c} \begin{array}{cc} & \sum_{i=\{x,y,z\}}{\left(\frac{\max\{\left(U\left(x\right)-U\left(x^{-d_{i}}\right)\right),\left(U\left(x\right)-U\left(x^{+d_{i}}\right)\right),0\}}{h_{i}}\right)}^{2}\\ & \;=\tilde{p}{\left(x\right)}^{2}, \end{array} \end{array}$$ (4)

where $x^{d_{i}}$ is the displacement of position **x** by one voxel in the *i*-direction, and *h*_*i*_ is the voxel size in dimension *i*. *U* is approximated using Eq 4 starting from **p**_0_ to **p**_1_ using a front propagation. To achieve this front propagation a controlled marching approach is used where every voxel position **x** is moving from three different sets: ALIVE: point for which *U* has been computed and frozen; TRIAL: point for which *U* has been estimated but not frozen; and FAR: point for which *U* is unknown. The method starts by including **p**_0_ in ALIVE, and assigning FAR the rest of points in the image. Then, in TRIAL are assigned the neighbor points of ALIVE belonging to FAR. In our case we use a 6-connected neighborhood. Then, *U* is estimated for the points in TRIAL. Further, in ALIVE is assigned the point in TRIAL with the lowest value of *U*. These steps are iterated until **p**_1_ ∈ *ALIVE*.

Subsequently, the minimum cost path is obtained by backtracking ∇*U*(**x**). To make the procedure more stable to noise, the vector field is normalized by: ∇*U*(**x**)_*N*_ = ∇*U*(**x**)/‖∇*U*(**x**)‖. ∇*U*(**x**)_*N*_ is set to zero in non-alive positions, then they do not affect the tracking. This backtracking is done by approximating the differential equation: ∂C*/∂*s* = −∇*U*(C*)_*N*_, starting from **p**_1_. This approximation is obtained using the fourth order Runge-Kutta method with a step size *δ* resulting in the optimal path $C_{p_{0},p_{1}}:R\to R^{3}$. In the rest of the paper we call the resulting centerline after applying Anisotropic Fast Marching (AFM) from **p**_0_ to **p**_1_ using cost $\tilde{p}$ as the result of the function *AFM*: $C_{p_{0},p_{1}}=AFM\left(\tilde{p},p_{0},p_{1}\right)$.

### 1.4 Cooperative centerline extraction

From three seed points **A**, **B**, and **C**, we get two minimum cost paths on $\tilde{p}$ using *AFM*: **A** → **B** ($C_{A}{}_{,B}=AFM\left(\tilde{p},A,B\right)$) and **A** → **C** ($C_{A}{}_{,C}=AFM\left(\tilde{p},A,C\right)$), where we would like to avoid intersections at some parts of the paths.

Inspired by [27], we propose a cooperative extraction of the paths **A** → **B** and **A** → **C**. The idea of this new method is to add to the cost $\tilde{p}$ a constraint around the neighbor centerline at the locations we do not want them to intersect, so the current path is influenced by the position of its neighbor. Ideally, we want to avoid overlap between the centerline and the neighbor artery; therefore the constraint region should cover this neighboring artery.

We start including the constraint region $\kappa \subseteq Z^{3}$ around the best path *C*^*Best*^ ∈ {*C*_**A,B**_, *C*_**A,C**_}, as we assume this path is not going to change much its position during the cooperative correction process. We define the best path as the one with the lowest average cost, where the total cost is given by the minimal action map *U* at the end point, which is divided by the path length to get the average cost. To constrain the neighbor path *C*^*N*^ to be at least a distance *ρ* from *C*^*Best*^, we include in $\tilde{p}$ a constraining region defined by a spherical dilation with radius *ρ* in all points of *C*^*Best*^ where we want to set the constraint. As we want to set the constraint after the artery bifurcation, the constraint region *κ* starts after this position where the artery starts bifurcating. Then after defining the constrain region *κ*, the new cost *p*_*N*_ is given by:

$$\begin{array}{c} p_{N}\left(x\right)=\{\begin{array}{c} \tilde{p}\left(x\right),\;if\;x\notin \kappa \\ K,\;if\;x\in \kappa \end{array}, \end{array}$$ (5)

where *K* is a high constant value. Therefore, the correction of path *C*^*N*^ is given by ${\tilde{C}}^{N}=AFM\left(p_{N},A,\left\{B,C\right\}\right)$.

**Fig 2:**
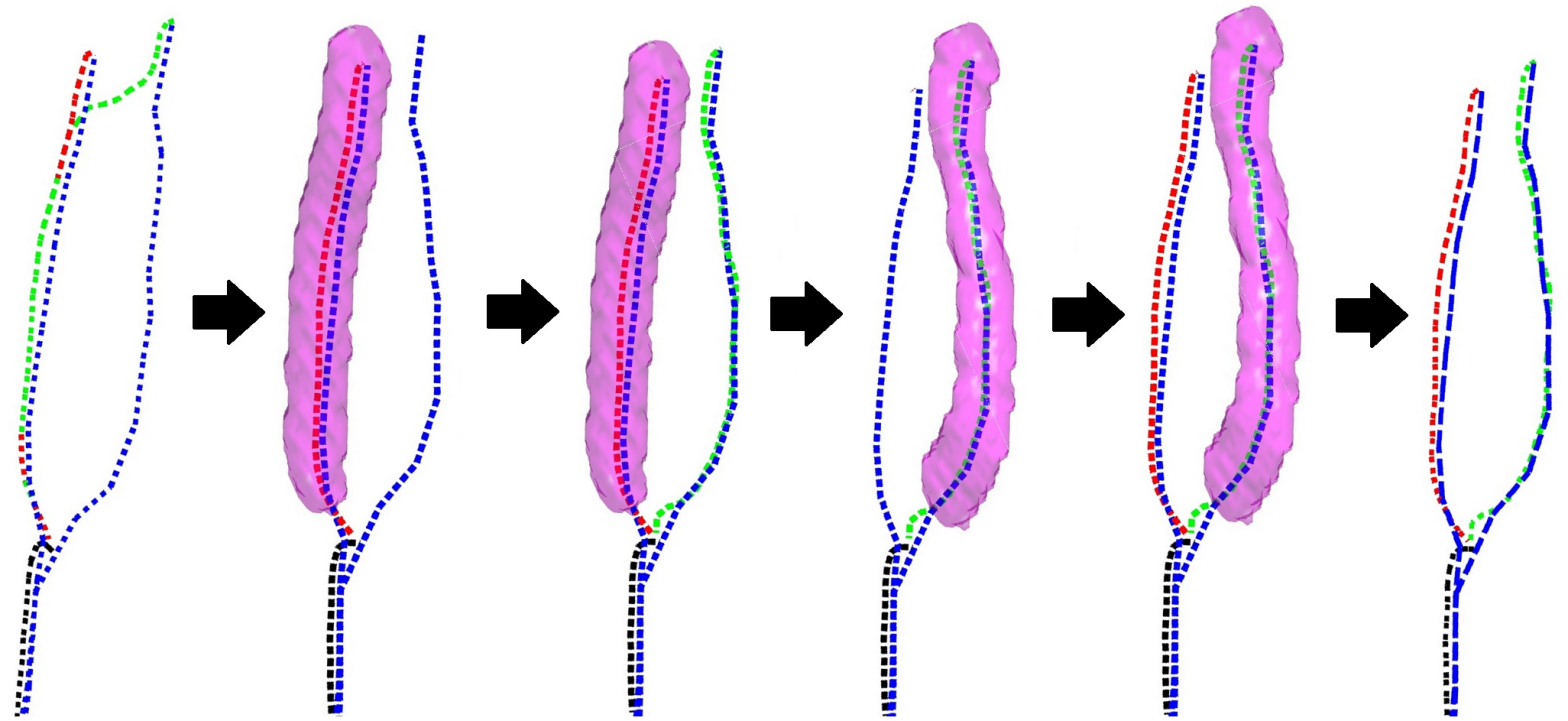
Schematic showing the cooperative centerline extraction method. Blue curves are the manually annotated centerlines; while black, red, and green curves are the automatic paths at CCA, ICA, and ECA respectively. First, the automatic paths are obtained using anisotropic fast marching, however the ICA and ECA erroneously follow the same path after the bifurcation. To fix this, a constraint region, shown by the purple surface, is constructed around the best path given by the red curve. Using this constraint, a new path is computed for ECA. Subsequently, a new constraint region is built around this new path. Then the path at ICA is computed again. Finally, both paths at ICA and ECA do not intersect and are close to the manually annotated ones.

Subsequently, a new constraint region around the corrected path ${\tilde{C}}^{N}$ is included in $\tilde{p}$ to correct *C*^*Best*^. This whole process is illustrated in Fig 2.

## 2 Experiments and results

### 2.1 Evaluated methods

From three marked seed points at CCA, ICA, and ECA; we cooperatively extract two paths between CCA-ICA and CCA-ECA. Here *κ* starts at the closest points of the initially estimated centerlines to an extra marked point at the artery bifurcation where the two arteries ICA and ECA separate. We call this Cooperative method from the Common to Internal and External CCIE. We also evaluated another cooperative approach where a centerline bifurcation point (BIF) is used as a seed point. BIF is a shifting of the artery bifurcation point (see Section 2.3). Using BIF as a seed point, we force the centerlines to pass through this point. Therefore, in this approach we extract three paths: between CCA-BIF, and cooperatively we extract two paths between BIF-ICA and BIF-ECA. We call this Cooperative approach between Common to Bifurcation to Internal and External CCBIE.

We compare the cooperative centerline approaches to the traditional approach to extract the centerlines independently. In one approach, two Separated independent centerlines are extracted from the Common to Internal and External (SCIE). And in other approach we use BIF as a seed point where three separated independent centerlines are extracted (SCBIE).

### 2.2 Image data

We used data from the Rotterdam study [30]. An MRI of the carotid bifurcation was performed in subjects with carotid artery plaque (defined as at least one carotid artery with a maximum wall thickness ≥2.5*mm* measured with ultrasound). The method was initially evaluated in a data set with manually annotated centerlines composed of 161 carotid arteries from 83 subjects. Five arteries had to be discarded due to manual annotation errors. Further, the method was evaluated in a data set of 3,904 arteries from 2,018 subjects where seed points were available but no manually annotated centerlines. Several MRI sequences were acquired: Proton Density Weighted Black-Blood MRI (BB), Proton Density Echo Planar Imaging MRI, 3D T1-weighted gradient echo MRI, T2-weighted Echo Planar Imaging MRI, and Phase Contrast MRI (PC). As in [1], BB and PC were used together to get the cost image *p* as described in Section 1.2, which are the sequences that provide a better description of the artery lumen [30]. The image resolutions are (in-plane voxel size × Slice thickness): 0.507 × 0.507 × 0.9*mm* for BB, and 0.703 × 0.703 × 1*mm* for PC.

### 2.3 Manual annotations

Manually annotated centerlines were obtained by an expert on the BB images using a similar annotation framework as described in [17]. Per artery, several points are annotated between CCA-ICA and between CCA-ECA to obtain two centerlines. These are further up-sampled using a cubic spline interpolation of resolution equal to the step size *δ* to get two higher resolution centerlines between CCA-ICA ($C_{ICA}^{M}$) and CCA-ECA ($C_{ECA}^{M}$). The seed points located at CCA (**x**_*C*_), ECA (**x**_*E*_), and ICA (**x**_*I*_) were obtained from the starting and end points of the manual centerlines, where **x**_*C*_ is the mean point between the two centerline starting points. In the experiments where manual centerlines are not available, the three seed points were manually placed in the BB images by an expert.

*Bifurcation point*:

Another point located at the artery bifurcation (**x**_*BIF*_) is manually annotated. This point is located at the gap between the two artery branches at the first slice starting from CCA where these two are visible. As for SCBIE and CCBIE require the bifurcation seed point at the centerline bifurcation, we had to shift **x**_*BIF*_ to be inside the vessel at the centerline bifurcation. To shift **x**_*BIF*_, we move it a certain distance through the path between **x**_*C*_- **x**_*BIF*_ ($C_{x_{C},x_{BIF}}$).

First, we define the estimated bifurcation $x_{BIF}^{e}$, which serves as ground truth of the centerline bifurcation in the images with manually annotated centerlines. This is obtained as the mean point between the first positions in $C_{ICA}^{M}$ and $C_{ECA}^{M}$ starting from **x**_*I*_ and **x**_*E*_ where the distance from these point positions to the neighbor centerline is below 1*mm*. We define the distance *D* from a point **x** to a path *C* by the *L*_2_ norm between the point and the closest point in the path:

$$\begin{array}{c} D(x,C)=\min_{y\in C}\left\|x-y\right\|. \end{array}$$ (6)

We verified by visual inspection that the resulting $x_{BIF}^{e}$ using this approach is close to the manual centerline bifurcation in most cases.

Subsequently, we define the path between the common point **x**_*C*_ and the annotated bifurcation **x**_*BIF*_ by $C_{x_{C},x_{BIF}}=AFM(\tilde{p},x_{C},x_{BIF})$. Then we find the closest point in the path to the estimated bifurcation $x_{BIF}^{e}$. This point represents the shifted bifurcation $x_{BIF}^{s}\in C_{x_{C},x_{BIF}}$. The distance between **x**_*BIF*_ and $x_{BIF}^{s}$ is the optimal shifting using this approach. The found optimal shifting for all 161 arteries was 5.1*mm* ± 1.6*mm*; therefore, we used *t* = 5.1*mm* to obtain $x_{BIF}^{s}$ in all cases.

### 2.4 Preprocessing

**Fig 3:**
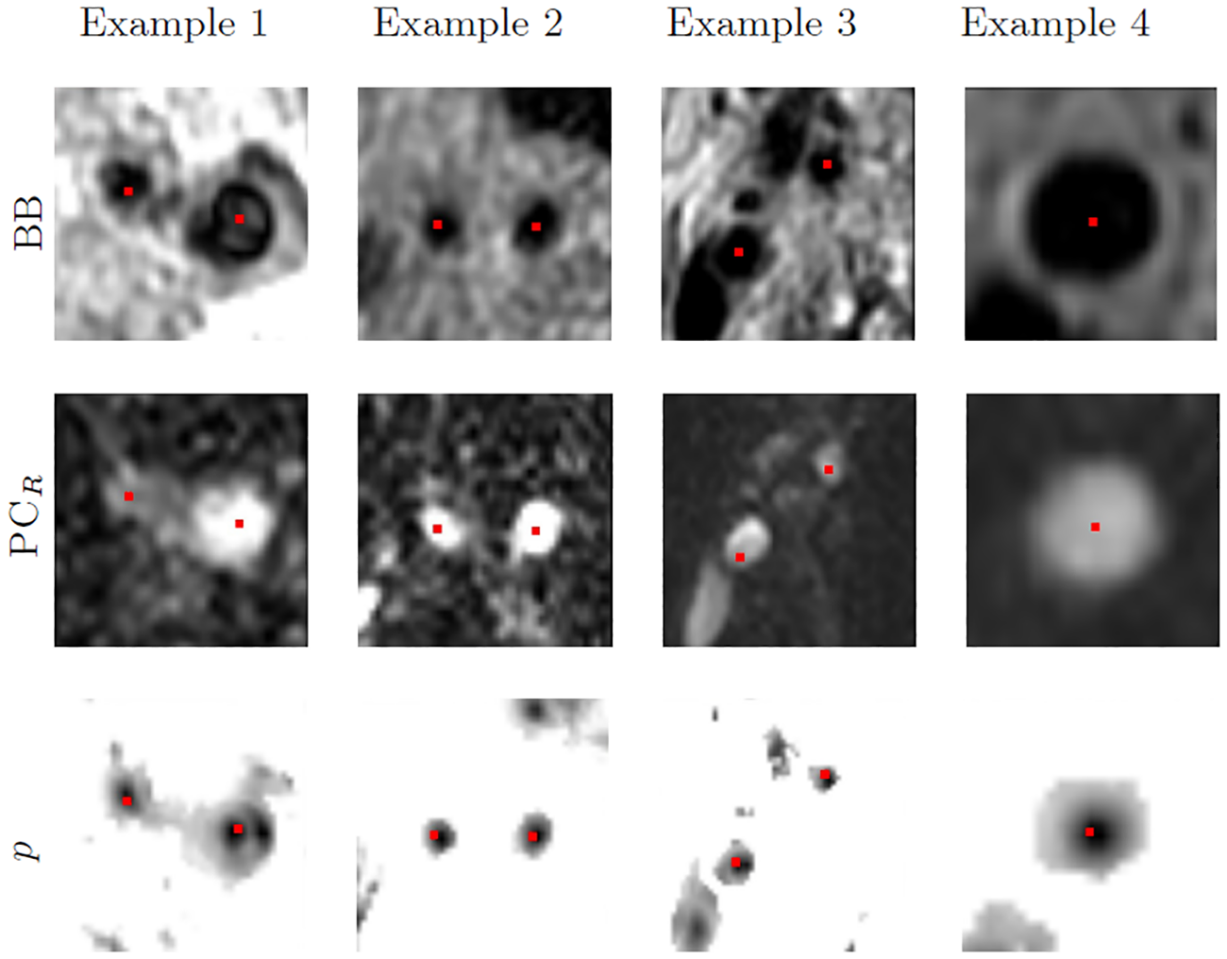
Examples of the registration and cost extraction results. BB images, registered PC to BB using the presented approach (PC_*R*_), and cost images (*p*) from four different arteries are shown. Manual centerlines overlaying the images are depicted in red.

The BB images suffer from intensity inhomogeneity [3]. This was corrected using N4 bias field correction [31], which is one of the most popular methods to correct intensity non-uniformity in MRI data. We used the default parameters of the method on the complete image as described in [31]. Further as in [1], PC images are registered to the BB images. However, by only using an affine registration as in [1], we observed several registrations errors that resulted in erroneous cost images *p*. We could obtain *p* using only one sequence, however as in [1] we also observed that it is useful to include both BB and PC to obtain *p*. Therefore, instead we used a different approach to perform the registration and include both sequences to get *p*. Similar to [4], we apply a rigid then a non-rigid registration using a registration mask. For the rigid registration, we use Euler transform, and for non-rigid a 3D B-spline transformation with 15 mm grid spacing, using in both mutual information as similarity metric. The registration mask must cover the artery in BB. For this a 10 mm diameter circular mask obtained by dilating the centerlines with a spherical structuring element with a radius of 5 mm is used. As this mask must roughly cover the artery, an accurate centerline is not needed. Therefore, to compute these centerlines we get two minimum cost paths between CCA(**x**_*C*_)-ICA(**x**_*I*_) and between CCA(**x**_*C*_)-ECA(**x**_*E*_) on a cost obtained from BB only (*p*_*BB*_). Then after registering PC to BB, we obtain the combined cost *p*. In Fig 3, the registered PC look well aligned, and the cost is low at the artery locations.

### 2.5 Parameter selection and configuration

As in [1], we choose the contribution of the medialness filter and lumen intensity similarity equal in Eq 1 (*α* = *β*). We evaluated several smoothing values *ω* ∈ {0, 1, 2, …10} to get $\tilde{p}$, where the selected value for each artery was obtained by leave-one-artery-out cross-validation. To make a faster computation of *U* and Δ*U*, the cost image $\tilde{p}$ is cropped in a minimum bounding box ±20 voxels in the x-y plane and ±3 voxels in the axial direction around the seed points. This bounding box size was enough to cover the artery in all cases. The step size *δ* is set to 0.1*mm*, which is significantly smaller than the voxel size.

The diameter 2 × *ρ* of the constraint region *κ* is set to 3.5*mm*. We observed this value was enough to cover the ICA and ECA artery lumen [32]. In CCIE and CCBIE, *κ* starts at the closest point of the initially estimated centerline to the artery bifurcation point **x**_*BIF*_.

### 2.6 Evaluation metric

To compare the automatically extracted centerlines to the manually annotated centerlines we compute the Hausdorff distance (*H*) between centerlines. The *H* distance between two centerlines *C*^*A*^ and *C*^*B*^ is defined as:

$$\begin{array}{c} H\left(C^{A},C^{B}\right)=\max\{\max_{x\in C^{A}}D\left(x,C^{B}\right),\;\max_{y\in C^{B}}D\left(y,C^{A}\right)\}. \end{array}$$ (7)

For each carotid artery two centerlines are defined for ICA and ECA. Thus the Centerline Artery Distance (CAD) between automatic ($C^{A}=C_{ICA}^{A}\cup C_{ECA}^{A}$) and manual artery centerlines ($C^{M}=C_{ICA}^{M}\cup C_{ECA}^{M}$) is given by the maximum distance between centerlines by:

$$\begin{array}{c} CAD\left(C^{A},C^{M}\right)=\max\{H\left(C_{ICA}^{M},C_{ICA}^{A}\right),\;H\left(C_{ECA}^{M},C_{ECA}^{A}\right)\}. \end{array}$$ (8)

We consider a centerline detection failed if the CAD between automatic and manual centerlines is above 3.5*mm*, as the mean artery radius is about this value [32], then the automatic centerline is likely to be outside the artery. We also confirmed the correctness of this CAD threshold by visually inspecting the position of the automatic centerlines with respect to the carotid artery.

### 2.7 Comparison with manual annotations

**Fig 4:**
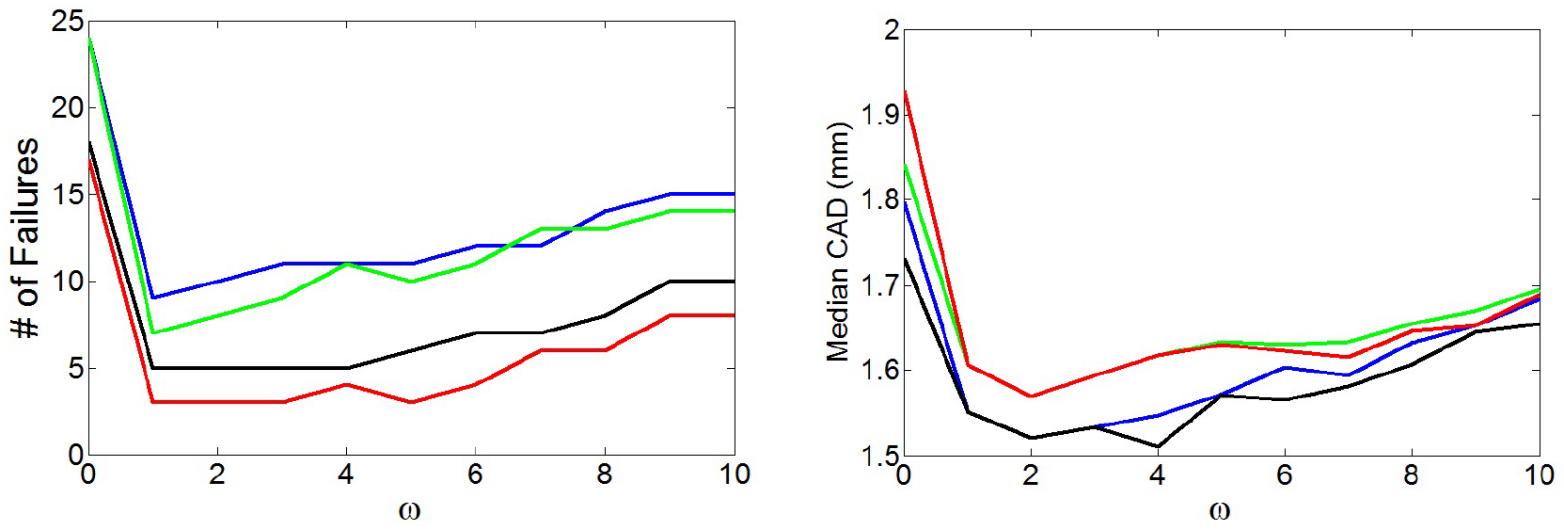
Number of failures and median CAD depending on the smoothing parameter *ω* for each method. SCIE is represented in blue, SCBIE green, CCIE black, and CCBIE red.

Initially, we observed the effect of the smoothing parameter *ω* in the different approaches. For each value of *ω*, we obtained CAD for all 161 vessels for each approach (see S1 Table). Median values and number of failures for each *ω* are shown in Fig 4. Better results were observed by enforcing some amount of smoothness in the centerlines. In all *ω* values, the cooperative centerline extraction methods (CCBIE and CCIE) showed better results than obtaining the centerlines independently (SCBIE and SCIE) in number of failures. A reduction in failures was obtained by including the bifurcation as an extra seed point (CCBIE Vs. CCIE and SCBIE Vs. SCIE), however this increases the median CAD slightly.

**Fig 5:**
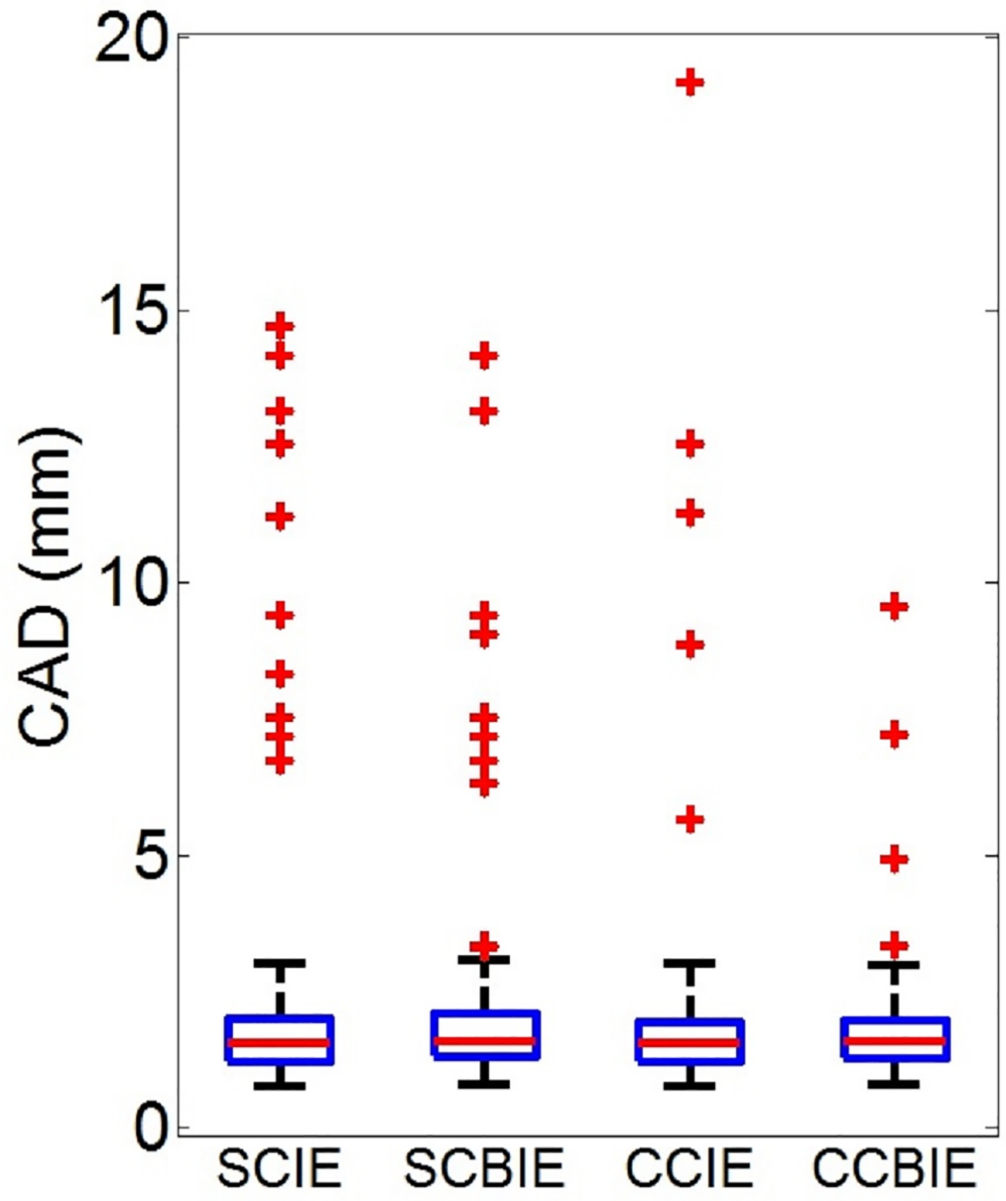
Box plot showing the CAD errors per method.

**Fig 6:**
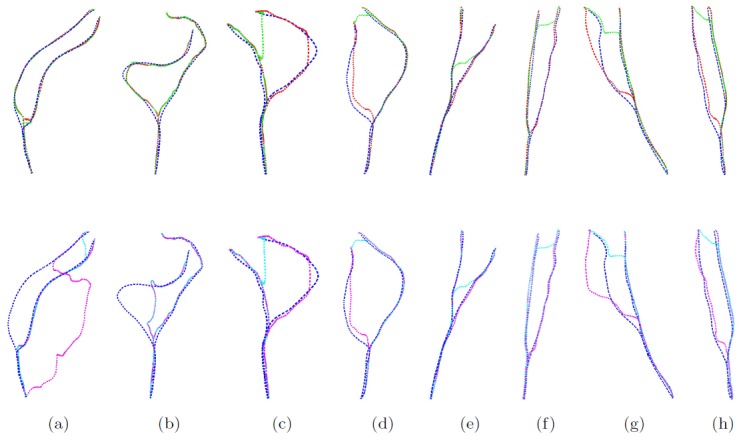
Examples of centerline results where failures are present. Manual centerlines are represented in blue, SCIE in cyan, SCBIE in green, CCIE in purple, and CCBIE red. Methods that use the bifurcation as a seed point are shown at the top row. SCIE fails in all cases (CAD error ≥3.5*mm*); SCBIE fails in cases c-h; CCIE fails in a, b, g; and CCBIE fails in g.

The optimal smoothing value *ω* per artery and for each method was selected by leave-one-artery-out cross-validation. From the training set of 160 arteries, we aimed to select the value for *ω* that resulted in least failures; in case several *ω* resulted in the same number of failures, the value resulting in the lowest mean CAD is selected. The number of failures per method are: SCIE: 10 failures, SCBIE: 8, CCIE: 5, and CCBIE: 3. A box plot describing the CAD results is shown in Fig 5. Significant results (McNemar test) for differences in the number of failures between methods is shown in Table 1. CCBIE is significantly better (*p* < 0.05) than SCIE. Other differences are not significant. Several centerlines examples using all methods are shown in Fig 6.

**Table 1:** Statistical significance of differences in number of failures between methods according to McNemar’s test. *p*-values are shown for each method comparison for the 161 arteries experiments described in Section 2.7 and for the 3,904 arteries experiment described in Section 2.8 (in parenthesis).

	SCIE	SCBIE	CCIE	CCBIE
SCIE	–	0.5	0.06	0.02
SCBIE	–	–	0.45	0.06(0.0001)
CCIE	–	–	–	0.5

### 2.8 Results in a large population data set

We also obtained the centerlines in a large data set from a population study composed of 3,904 arteries from 2,018 patients. We compare the best method to extract the centerlines independently (SCBIE) to the best method to extract them cooperatively (CCBIE). However, for this data set we do not have manually annotated centerlines, but only the seed points at CCA, ICA, ECA, and the bifurcation. Therefore, to compare the two methods, we first compute CAD between the two centerlines (see S2 Table). Then, we looked for large differences so centerlines with CAD above 3.5*mm* are the cases of interest. From the 3,904 arteries, we found 42 to have CAD between SCBIE and CCBIE above 3.5*mm*. A visual inspection of these 42 cases revealed that in 28 cases CCBIE is correct while SCBIE fail, 4 SCBIE are correct while CCBIE fail, in 7 cases both fail, and in no case both are correct. One case was discarded due to wrong seed point locations, and due to occlusion of arteries, the centerline could not be reliably assessed in two arteries. Applying McNemar test, we obtain that CCBIE is significantly better than SCBIE (*p* < 0.0001) resulting in fewer centerline failures.

## 3 Discussion

In this work we presented a method for improved extraction of the carotid artery centerlines which results in fewer failures. With this method both artery centerlines from CCA to ICA and ECA are extracted cooperatively by integrating geometrical information of the artery bifurcation in the cost. Inspired by [27], geometrical information is integrated as constraint sections around the paths to prevent path intersections. We demonstrated that the presented method performs better than the traditional approach that extracts the centerlines independently.

Commonly the centerlines in the carotid artery bifurcation are extracted as two minimum cost paths [1, 13–16], however if the cost image is not well defined failures may arise. In this work, we showed that these inaccuracies in the cost could be overcome using the presented cooperative centerline extraction approach. Another possible solution to get better centerlines would be to improve the cost image. In [1, 33], a refined cost is generated using the extracted centerline, where the medialness is calculated at planes sampled perpendicular to the centerline, which may result in a better cost image. However, this approach is likely to fail if the centerline used to reformat the image is outside the vessel lumen. Therefore, the failure cases addressed in this work would not be prevented using this approach. For instance in [1], using this approach they reported two failed cases out of 76 arteries and discarded two other arteries due to bad image quality. This is a similar performance to our approach to extract two independent centerlines SCIE (4 fails out of 78 Vs. 10 fails out of 161).

There are other methods to jointly or cooperatively extract the centerlines based on evolution approaches [24], skeletonization of the segmentation [25], and matching appearance models [26]. Evolution approaches require an initialization, where if this is outside the artery an incorrect centerline would be likely obtained, as these methods may get stuck in a local minimum. Skeletonization of the segmentation may work well in cases of relatively correct segmentations, however segmentation of the carotid artery from MRI is not an easy task and most methods require a centerline as initialization. Skeletonization of segmentations using the method presented in [34] may be a good option, as it does not require the centerline as initialization and presents good results. The matching appearance model presented in [26] is an interesting method as it is fully automatic, however this could fail in complex geometries. In any of these methods it is not guaranteed that the vessels do not intersect after the bifurcation which is a common source of centerline failures, while in the presented method we guarantee both centerlines will not follow the same path after the bifurcation.

Another possible solution to get more accurate centerlines is applying smoothness constraints to the path, as the path intersection errors are often accompanied by high curvature paths. We explored this solution in Section 2.7 by adding a constant *ω* to the cost image similar to [21], which penalizes long paths, and therefore reduce curvature. We proved the smoothing to be useful as fewer failures and more accurate centerlines were obtained when applying a certain level of smoothing. We also observed that high smoothing values affected the results negatively. Even though overall the smoothing improves the centerlines, still several failures were obtained in SCIE. Therefore, smoothing alone is not enough to significantly reduce centerline failures.

Using the cooperative approaches (CCIE and CCBIE) we showed better results in terms of number of failures than using the approaches without interaction between paths (SCIE and SCBIE). Additionally, we observed that only including the bifurcation point as an extra seed point in SCBIE seemed to have already a small positive effect in terms of number of failures compared to SCIE. However this effect was not significant in the 161 arteries set. Using the cooperative centerline extraction methods, we also showed a slight reduction of number of failures by using the bifurcation as an extra seed point in CCBIE instead of a reference point to build the constraint regions as in CCIE. Fig 6 showed that the centerlines in CCIE may fail before the artery bifurcation, so adding the extra seed point helps to prevents these failures.

However, Fig 4 seems to indicate that adding the bifurcation as extra seed point also reduces the accuracy of the centerlines in non-failure cases. This can be explained partly by the fact that in our data, the annotated bifurcation point is located at the artery bifurcation which is easy to visualize, then this point had to be shifted to be used as a seed point, which may introduce small inaccuracies in the centerlines. Despite the accuracy reduction in non-failure cases, we believe CCBIE to be the preferred method because it results in the smallest number of failures.

Even though the cooperative approach leads to fewer failures, a disadvantage is that it requires one additional annotated point. However, we argue one point is not too much work to annotate. Additionally, there are methods to automatically extract bifurcation points which could potentially be used in the carotid artery [35]. Therefore, we think using an extra annotated point at the bifurcation results in a positive trade-off.

Another disadvantage of the proposed cooperative method compared to the approach to extract the centerlines independently is the need to perform two extra minimum cost path computations. However, algorithms with logarithmic complexity for fast AFM computation are available [20], so the computations of extra paths should not represent a big issue in most current processing machines.

The best method CCBIE, still resulted in three failures out of 161 arteries and 11 failures out of 42 difficult cases in the larger study. We observed the cost images to be poorly defined in some cases due to registration errors, and in other cases neighbor structures close to the artery had a lower cost. An improved registration, and cost extraction which can discriminate neighbor structures could be investigated.

For this study we used moderately diseased patients data, for future work an evaluation including highly diseased patients would be recommended. However, we would expect the presented method to work well in these cases as we observed good centerlines in the diseased sections of the arteries.

The main reason to extract the carotid artery centerline is to define a ROI for further processing such as registration [4], or to initialize a segmentation method [1–3], where reducing the centerline failures is very important. If the centerlines follow a wrong path outside the vessel, a registration or segmentation will likely fail [2, 5]. Therefore, we think the presented method to extract the centerlines is highly suitable for further processing, as it considerably reduces the number of failures. Additionally, this method might be applicable in other applications as neuron tracking, pulmonary tree extraction, coronary centerlines; as this method could be easily extended to extract more than two centerlines.

## 4 Conclusion

In conclusion, we present a simple, yet effective approach to improve centerline extraction in the carotid artery bifurcation, which significantly reduced the number of centerline tracking failures.

## Supporting information

S1 Table Methods results. Distances between the automatic centerlines obtained using the presented methods and the manual centerlines per artery and per *ω* in the 161 arteries data set.
(XLS)

S2 Table Results in large population data set. Distances between the automatic centerlines obtained using the methods SCBIE and CCBIE per artery in the large population data set.
(XLS)
